# Effect of a lifestyle-focused electronic patient support application for improving risk factor management, self-rated health, and prognosis in post-myocardial infarction patients: study protocol for a multi-center randomized controlled trial

**DOI:** 10.1186/s13063-018-3118-1

**Published:** 2019-01-24

**Authors:** Manuel Gonzalez, Ingela Sjölin, Maria Bäck, Halldora Ögmundsdottir Michelsen, Tina Tanha, Camilla Sandberg, Alexandru Schiopu, Margret Leosdottir

**Affiliations:** 1 Heart Center and Department of Public Health and Clinical Medicine, Cardiology, Umeå University, Umeå, Sweden; 2 Commonwealth Scientific Research and Industrial Organisation (CSIRO), Brisbane, Australia; 3 Department of Clinical Sciences Malmö, Lund University, Malmö, Sweden; 4 Department of Cardiology, Skane University Hospital, 205 02 Malmö, Sweden; 5 Department of Medical and Health Sciences, Division of Physiotherapy, Linköping University, Linköping, Sweden; 6 Department of Occupational Therapy and Physiotherapy, Sahlgrenska University Hospital, Gothenburg, Sweden; 7 Department of Community Medicine and Rehabilitation, Physiotherapy Umeå University, Umeå, Sweden

**Keywords:** eHealth, Myocardial infarction, Cardiac rehabilitation, Web-based application, Smartphone application, Cardiovascular, Risk factors, Prognosis, Quality of life

## Abstract

**Background:**

Cardiac rehabilitation (CR) programs addressing risk factor management, educational interventions, and exercise contribute to reduce mortality after myocardial infarction (MI). However, the fulfillment of guideline-recommended CR targets is currently unsatisfactory. eHealth, i.e., the use of electronic communication for healthcare, including the use of mobile smartphone applications combined with different sensors and interactive computerized programs, offers a new array of possibilities to provide clinical care. The present study aims to assess the efficacy of a web-based application (app) designed to support persons in adhering to lifestyle advice and medication as a complement to traditional CR programs for improvement of risk factors and clinical outcomes in patients with MI compared with usual care.

**Methods/design:**

An open-label multi-center randomized controlled trial is being conducted at different CR centers from three Swedish University Hospitals. The aim is to include 150 patients with MI < 75 years of age who are confident smartphone and/or Internet users. In addition to participation in CR programs according to the usual routine at each center, patients randomized to the intervention arm will receive access to the web-based app. A CR nurse reviews the patients’ self-reported data twice weekly through a medical interface at the clinic. The primary outcome of the study will be change in submaximal exercise capacity (in watts) between 2 and 4 weeks after discharge and when the patient has completed his/her exercise program at the CR center, usually around 3–6 months post-discharge. Secondary outcomes include changes in self-reported physical activity, objectively assessed physical activity by accelerometry, self-rated health, dietary, and smoking habits, body mass index, blood pressure, blood lipids, and glucose/HbA1c levels between inclusion and follow-up visits during the first year post-MI. Additionally, we will assess uptake and adherence to the application, the number of CR staff contacts, and the incidence of cardiovascular events at 1 and 3 years after the MI. Patient recruitment started in 2016, and the first study results are expected in the beginning of 2019.

**Discussion:**

The present study will add evidence to whether electronic communication can be used to improve traditional CR programs for patients after MI.

**Trial registration:**

ClinicalTrials.gov, NCT03260582. Retrospectively registered on 24 August 2017.

**Electronic supplementary material:**

The online version of this article (10.1186/s13063-018-3118-1) contains supplementary material, which is available to authorized users.

## Background

During the last decades, mortality rates from coronary heart disease (CHD) have decreased, with more than 50% of the mortality reduction attributable to better control of traditional cardiovascular risk factors on a population level (primary prevention) [1, 2]. Regarding the treatment of established disease, secondary prevention administered through cardiac rehabilitation (CR) is the major contributor to the mortality reduction [3]. CR includes specific core components comprising baseline patient assessment, nutritional counseling, risk factor management, psychosocial interventions, physical activity counseling, and exercise [4].

It is well documented that participation in CR programs improves risk factor control and therapy adherence, enhances quality of life, and reduces recurrent events [5, 6]. However, the incomplete fulfillment of guideline-recommended CR targets is currently a matter of concern [7]. In the latest annual report from the Swedish Web-System for Enhancement and Development of Evidence-Based Care in Heart Disease Evaluated According to Recommended Therapies (SWEDEHEART) registry, it was shown that at 1 year after a myocardial infarction (MI) only 21% of patients reached the four main treatment objectives: abstinence from smoking, systolic blood pressure < 140 mmHg, low-density lipoprotein cholesterol < 1,8 mmol/L, and active participation in a supervised exercise program as a part of CR [8]. One main barrier to target achievement is limited accessibility to CR programs [9]. Also, while international recommendations advocate program flexibility and individual tailoring, most of the current CR programs are rigid and time-limited and demand substantial healthcare resources [9, 10]. Therefore, all main international heart associations have claimed for the reengineering of CR to enhance access, adherence, and effectiveness. The general call is for the development of innovative and cost-effective CR programs that are oriented to modify lifestyle and behavior with sustainable results and may be easily integrated into the pre-existing healthcare structures [9, 10]. eHealth, i.e., the use of electronic communication and information technologies in healthcare, offers a whole new array of possibilities to provide clinical care. These include, for example, distance monitoring via telecommunication and sensors, interactive computer programs, and smartphone applications. While there are thousands of available eHealth applications on the market, only a small minority have been tested in a controlled manner with proper guidance from healthcare personnel. This is crucial, as investment in new treatments and technologies in healthcare needs to be evidence-based [11, 12]. The few published studies on eHealth solutions in CR, mostly using Internet and smartphone-based interventions, have reported enhanced self-management of risk factors with indications that the interventions have the potential to improve well-being, decrease risk for recurrent events, and increase adherence to medication [13–17]. However, most of these studies have included only small numbers of patients with short follow-ups (weeks or months) and have investigated a reduced number of surrogate endpoints, limiting clinically useful conclusions [18].

### Aim of the study

The study will assess the efficacy of a web-based application as a complement to traditional CR programs for improvement of secondary prevention outcomes in post-MI patients, compared with usual care. The hypothesis is that the intervention will enhance patient adherence to lifestyle advice (exercise, daily physical activity, healthy diet, and tobacco abstinence) and medication, resulting in better risk factor control and prognosis as well as improved self-rated health.

## Methods/design

### Study design, setting, and patient characteristics

**Fig. 1:**
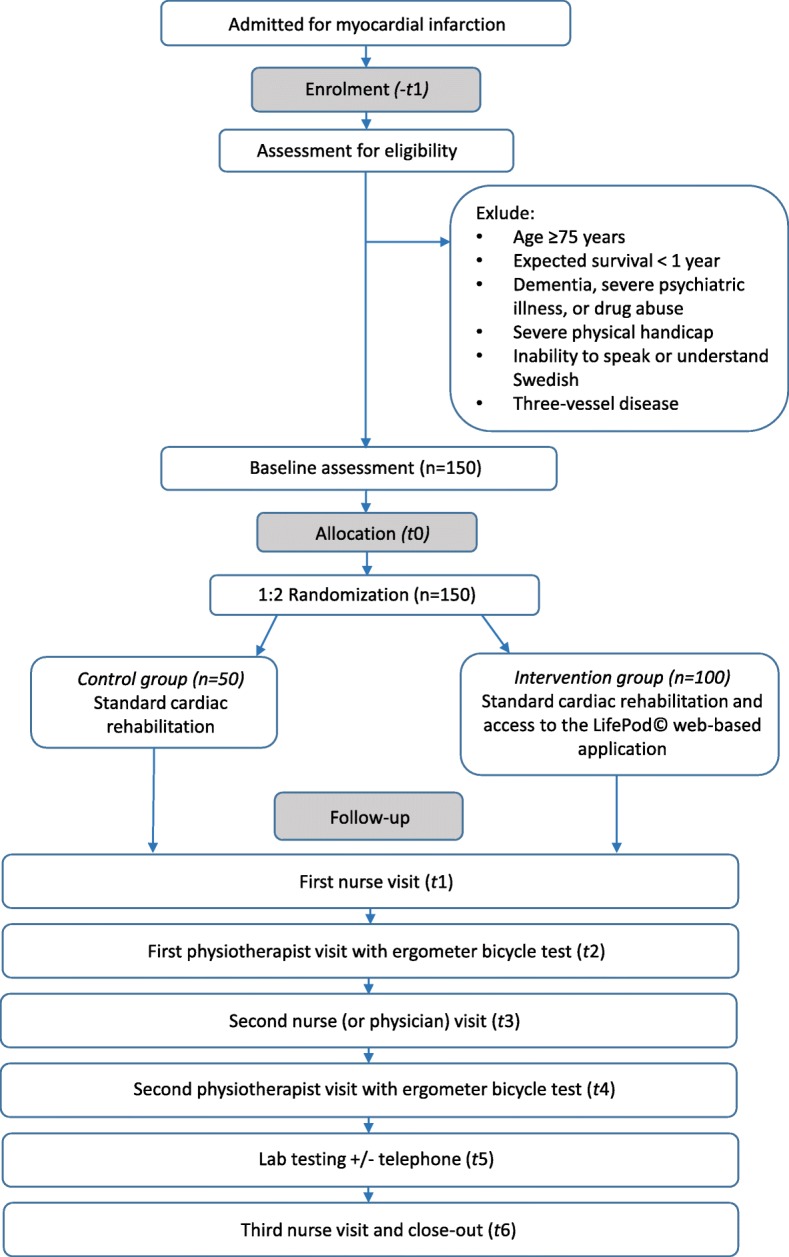
Flow diagram for the study

The study is an open-label multi-center randomized controlled trial. A total of 150 patients will be recruited from three CR centers based at university hospital clinics in Sweden (Fig. 1). Two of these are in the densely populated Skåne region (southern Sweden), while one is situated in the rural area of Norrland (northern Sweden). In total, approximately 1200 patients < 75 years of age are treated for acute MI at the three study centers each year. These patients will form the basis for the project cohort. Based on our feasibility study (see details in a subsequent section), we estimate that approximately 35% of patients screened for eligibility will be included in the study. As screening will not be done during weekends, holidays, or vacation high seasons, the recruitment is projected to take at least 1.5 years.

Patients will be screened for eligibility and offered participation in the study within 2 weeks of their index event (MI), preferably while still being treated in the coronary care unit (CCU). Specially trained research nurses and physiotherapists will be responsible for the recruitment process.

#### Inclusion criteria

The study inclusion criteria are the following:

- Age < 75 years. This cut-off is set as only those < 75 years of age are followed in the national Secondary Prevention after Heart Intensive Care Admission (SEPHIA) registry, which is a part of the Swedish heart registry SWEDEHEART.
- Patient has suffered an MI within the last 2 weeks
- Patient owns a smartphone and/or has access to Internet via a computer or tablet (surf pad) and can handle the software

#### Exclusion criteria

The following are the exclusion criteria:

- Expected survival < 1 year
- Dementia, severe psychiatric illness, or drug abuse
- Severe physical handicap limiting the patient’s ability to participate in an exercise-based CR program
- Inability to speak or understand the Swedish language
- Three-vessel disease requiring coronary artery bypass grafting

### Randomization

Randomization will be done using sealed envelopes containing information as to which arm the patient is randomized. The envelopes will be prepared by a member of our research team and subsequently mixed by another member of the team. Based on power calculations, the estimated sample size is 50 patients in each arm (for details, see the section on Statistical analysis, power, and sample size). However, due to expected loss of adherence in the intervention arm, we decided to double the size of the intervention arm. As such, 50 patients will be randomized to the control arm and 100 patients to the intervention arm.

### Usual care

**Fig. 2:**
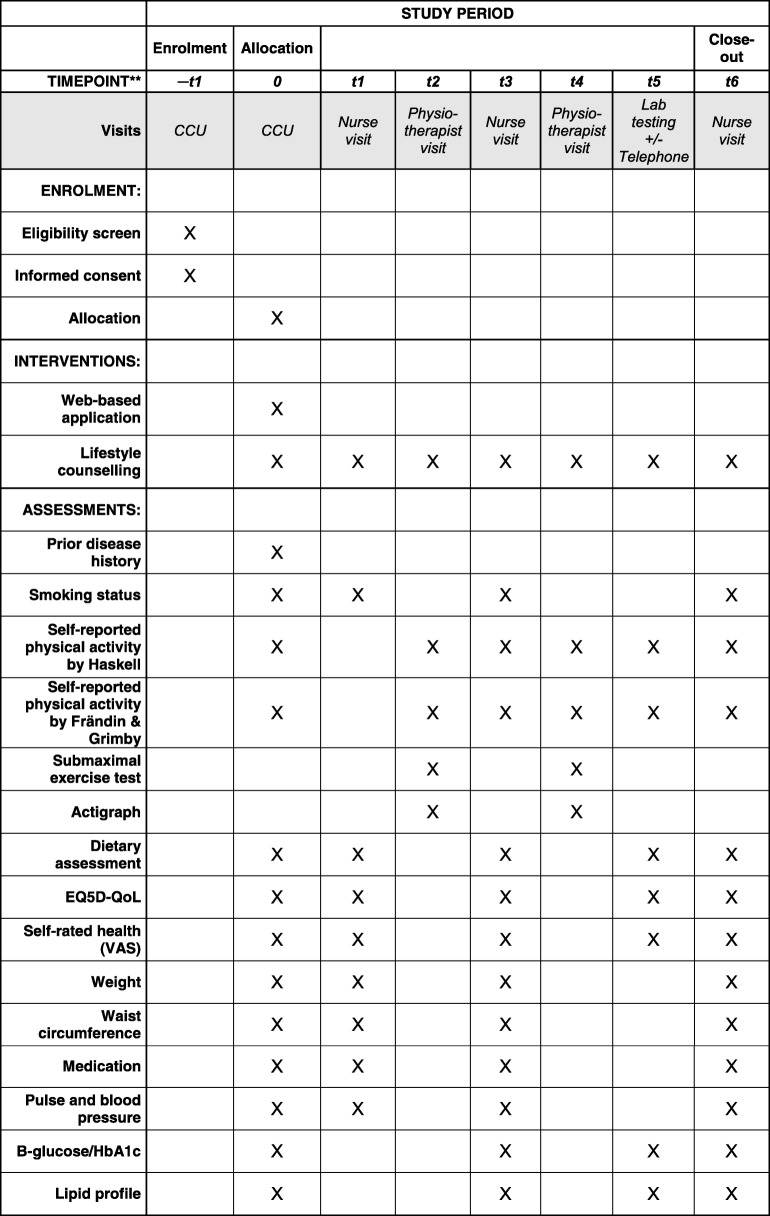
Standard Protocol Items: Recommendations for Interventional Trials (SPIRIT) figure for the study. Lifestyle counseling and assessments are done for all patients, i.e., in both the control and the intervention arms. *CCU* coronary care unit, *t*1 first nurse visit (2–4 weeks after the index event), *t*2 first physiotherapist visit (2–4 weeks after the index event), *t*3 second nurse visit (6–10 weeks after the index event), *t*4 second physiotherapist visit (4–6 months after the index event), *t*5 measurement of serum lipids and glucose/HbA1c (6–8 months after the index event) followed by a telephone call in case of abnormal test results, *t*6 third nurse visit (conducted 12–14 months after the index event)

All patients, in both the control arm and the intervention arm, will be offered participation in CR programs according to the usual routine at each center. The CR programs at the three study centers have approximately the same follow-up protocol, as they all report to the SEPHIA registry, which requires data registration at fixed time points. Minimally, the CR program includes five out-patient visits at the CR center. The patient’s first contact is with a CR nurse within 2–4 weeks from discharge (*t*1), closely followed by a first physiotherapist visit (*t*2) at which a symptom-limited bicycle ergometer test is performed (Fig. 2). A second nurse or physician visit (*t*3) is offered at 6–10 weeks post-discharge. A follow-up visit with a physiotherapist (*t*4) is offered when the patient has completed his/her exercise program at the CR center, usually around 3–6 months post-discharge. A second ergometer test is performed at the *t*4 visit. At 6–8 months, laboratory measures of lipids and glucose/HbA1c are performed (*t*5), followed by a telephone call in case of abnormal results. A last visit to a nurse (*t*6) is offered at 12–14 months after the index event. Information on lifestyle (exercise, daily physical activity, diet, and smoking), risk factor levels (blood pressure, weight, waist circumference, fasting plasma glucose, Hb A1c, serum lipids), and self-rated health (measured with the EQ-5D quality of life (QoL) questionnaire and a general self-rated health visual analog scale 0–100) are controlled at the nurse and physiotherapist visits according to Fig. 2 [19].

All patients will be offered participation in the traditional CR program at each study center irrespective of whether they drop out of the current study (both arms) or do not fully engage in the study intervention (intervention arm).

### The intervention

Patients randomized to the intervention arm will additionally receive access to the LifePod® support software, a web-based application designed to support persons in adhering to lifestyle advice and medication. LifePod® consists of two main parts, a patient interface (a web-based application) and a medical interface managed by healthcare professionals.

#### The patient interface

**Fig. 3:**
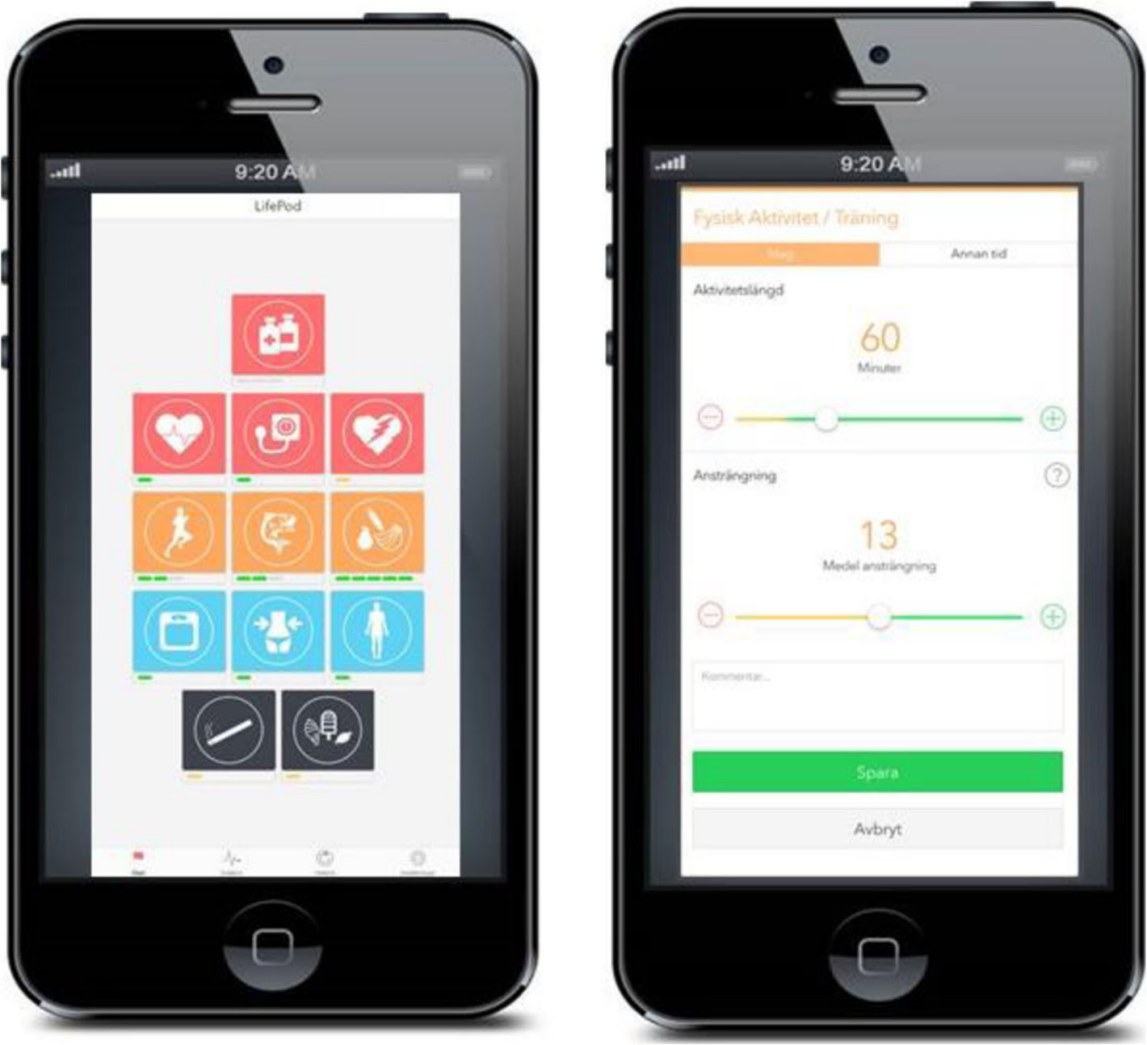
Screenshots of the LifePod® patient interface. The patient interface can be accessed through a smartphone, tablet or PC

The patient interface is a web-based application (app) accessible through a smartphone, computer, or tablet, where the patient can log information about his/her lifestyle (i.e., diet, exercise, and smoking), measurements (i.e., weight, pulse, and blood pressure), symptoms, and medication. Screenshots of the patient interface can be seen in Fig. 3. The patient can review his/her data in graphs displaying registered values in relation to guideline-recommended targets. The software provides direct positive feedback on healthy choices as well as giving general recommendations on exercise, daily physical activity, and healthy diet. Additionally, the patient receives notices when the nurse at the clinic has reviewed his/her profile. The software generates reminders in case of decreasing registrations or missed medication. Finally, short text messages (SMS) will be sent out two to three times a week with information and tips on a healthy lifestyle. The interface can be customized, turning lifestyle/measurement options and reminders on or off depending on the needs and preferences of each patient. During admission, if the patients in the intervention arm do not have their own smartphones available, tablets will be provided to enable them to get acquainted with the software.

#### The medical interface

**Fig. 4:**
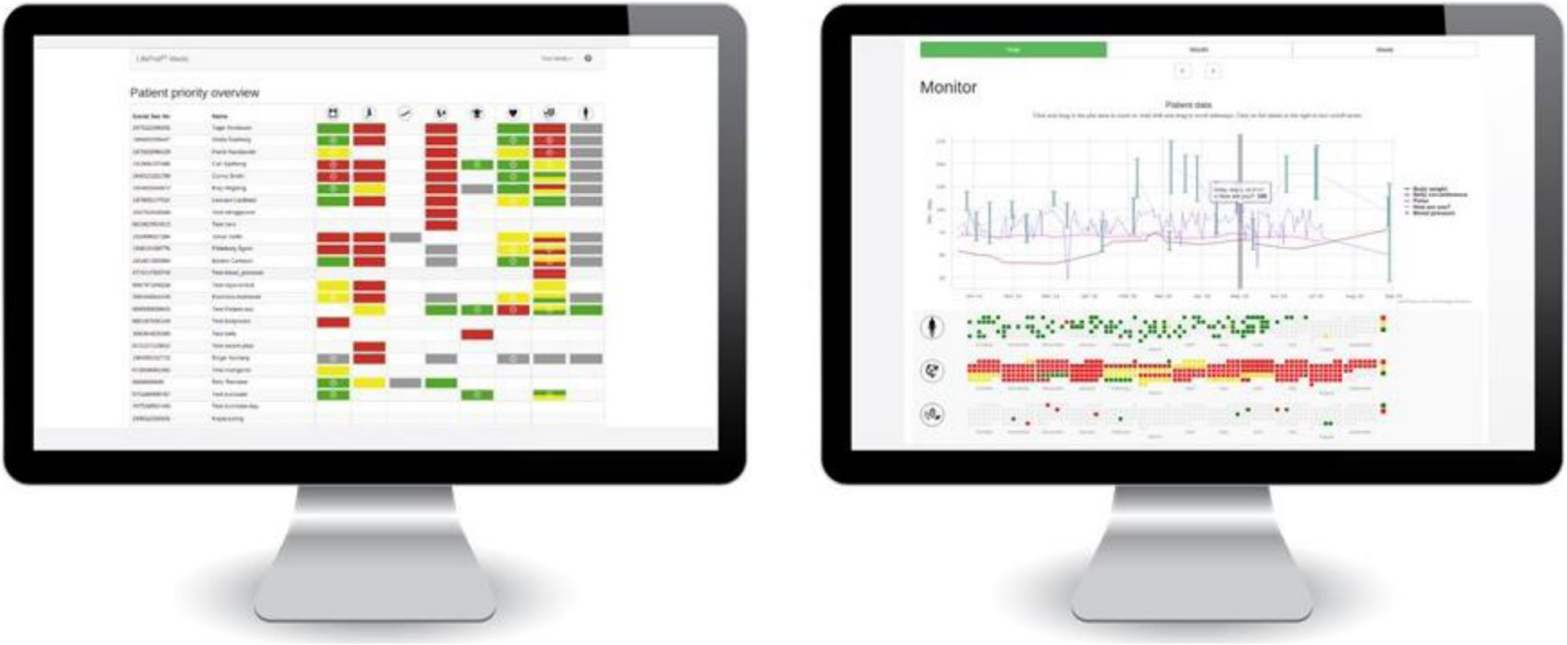
The LifePod® medical interface

All information that the patients log in the app is transferred to a medical interface that can be accessed by the treating nurses, physiotherapists, and physicians at the CR center. Additionally, the system ranks the patients, giving high priority for example to patients reporting chest pains, not taking their medications, or who are lagging behind with their exercise regime, while patients that follow recommendations and are doing well receive a lower ranking (Fig. 4). This information is reviewed by the CR nurse twice weekly. The algorithm in the LifePod® system thus gives priority support for the healthcare professional based on the patients’ reported outcomes. This allows for targeted interventions and better resource management. In addition, the system gives the caregiver the opportunity to manage and guide both individual patients (1:1 follow-up) and large patient populations (1:1000 or more).

### Data collection and follow-up

For the current study, baseline data will be retrieved from Sweden’s Register of Information and Knowledge about Swedish Heart Intensive Care Admissions (RIKS-HIA ), which, like SEPHIA, is part of SWEDEHEART. RIKS-HIA follows the quality of the acute care of MI patients. Baseline data will include age, prior disease history and medication, type of MI, medical treatment, and laboratory and physiological measures. In addition to variables included in RIKS-HIA, all patients will be asked to fill out questionnaires on diet, physical activity, and self-rated health at baseline. Follow-up data will be retrieved from the SEPHIA registry.

### Outcomes

#### Primary outcome

The primary outcome, which the power calculations are based on, is change in submaximal exercise capacity (in watts, W) between the first and second physiotherapist visits. The submaximal exercise capacity, which reflects the patients’ level of physical fitness, is evaluated at two physiotherapist visits, the first at 2–4 weeks after discharge and the second when the patient performs the exercise program at the CR center, usually around 3–6 months post-discharge (Fig. 2). The test is performed on a bicycle ergometer according to the World Health Organization (WHO) protocol, with an increased workload of 25 W every 4.5 min [20, 21]. The initial starting load, 25 W or 50 W, is decided based on the patient’s exertion history. After 2 and 4 min of each workload, heart rate, perceived exertion according to Borg’s rating of perceived exertion (RPE) scale, and subjective symptoms, including chest pain and dyspnea according to Borg’s Category Ratio (CR-10) scale, are registered [22]. After 3 min, the systolic blood pressure is measured. The exercise test is discontinued at Borg RPE 17 and/or dyspnea 7 on Borg’s CR-10 scale.

#### Secondary outcomes

The secondary outcomes are described as follows:

1. Changes in the following variables measured from inclusion to first, second, and third nurse visits, respectively:
  - Self-reported health using the visual analog scale (see Additional file 1)
  - Healthy diet index as evaluated within SEPHIA (see Additional file 1)
  - Smoking habits (self-reported)
  - Weight (kilograms), body mass index (BMI, in kilograms/meter^2^), and waist circumference (in centimeters)
  - Systolic and diastolic blood pressure (mmHg)
  - Total cholesterol, low-density lipoprotein and high-density lipoprotein cholesterol, and triglycerides in plasma
  - Fasting plasma glucose and HbA1c
2. Changes in self-reported physical activity (by Haskell and Frändin and Grimby; see Additional file 1) measured from inclusion to first and second physiotherapist visits, respectively [23, 24]
3. Uptake (proportion of patients who log on to the patient interface at least once) and adherence (proportion of patients registering data at least twice per week on a weekly basis throughout the intervention period)
4. Number of telephone and physical contacts with the CR staff
5. Incidence of cardiovascular events at 1 and 3 years after the index event: hospitalization for a new MI, heart failure or stroke, and cardiovascular death
6. Correlations between self-reported physical activity levels and objective measurements of physical activity and sedentary time measured by a validated instrument for 7 consecutive days, as analyzed using ActiGraph accelerometers. ActiGraph accelerometers are widely used in research because they have shown high validity and reliability [25, 26]. The measurements will be performed in conjunction with the first and second physiotherapist visits (*t*2 and *t*4) on patients recruited at Skåne University Hospital in Malmö (*n* = 60).

As recruitment was finalized in April 2018 and patients are followed for 1 year, the final study results will first be available by the fall of 2019.

### Data management and monitoring

Primary responsibility for data monitoring will be assumed by the principal investigator (PI) and local study coordinators. The PI will oversee all data sharing, data integrity, and data security. The PI takes primary responsibility for ensuring that the design of the study meets appropriate standards and that arrangements are in place to ensure appropriate conduct and reporting. The trial will be run in accordance with Good Clinical Practice (GCP), and all data will be handled according to the Swedish Data Protection Authority and General Data Protection Regulations (GDPR).

### Feasibility study

We performed a non-randomized feasibility study to test the LifePod® application as a partial alternative to standard care, by recruiting 50 patients between September 2013 and January 2015. Results showed that 35% of the screened patients were included in the study. The main reasons for non-participation were lack of interest (41%), inadequate Swedish language skills (27%), and inability to manage the software (23%). Uptake, measured as the percentage of patients who logged on to the patient interface at least once, was 40%. Adherence during the first 6 weeks, measured as the percentage of patients registering data at least twice a week on average during weeks 1–2, 3–4, and 5–6, was 82%, 63%, and 56%, respectively. The main reasons for poor uptake and loss of adherence were finding the software too complicated, new illness, lack of time (especially after returning to work), and loss of motivation. Patients called for a more user-friendly interface, more positive feedback, and implementation of sensors. The preliminary results from the feasibility study have been used to improve the software. The upgraded version is more simple and user-friendly; reminders have been implemented, patient feedback has been boosted, and short messages with information on healthy lifestyle have been added. The medical interface has also been improved according to wishes from healthcare personnel involved in the feasibility study.

### Statistical analysis, power, and sample size

Sample size calculations were based on results from a local registry (SEPHIA) data analysis including 40 post-MI patients attending CR at one of the study centers (Skåne University Hospital in Malmö). Out of the 40 patients included in the analysis, 73% participated in a hospital-based exercise program, on average 7 times (range 2–12) during an 8-week exercise period. The difference (Δ) in cardiac exercise capacity (W), measured using a symptom-limited bicycle ergometer test at 2–4 weeks post-discharge and after completing a hospital-based exercise program (around 3–6 months post-discharge), was calculated. The average difference was + 14.6 (± 15.6) W. With a power of 90%, a two-sided significance level of 0.05, and a least mean difference at 10 W (standard deviation (SD) ± 20 W) between groups, the estimated sample size was 150 patients: 50 patients in the usual care arm and 100 patients in the intervention arm. This included an intention-to-treat dilution effect based on a 40% loss of adherence in the intervention arm, an estimation based on our own feasibility study as well as other studies reporting adherence to eHealth interventions in CR [11, 13–15, 17]. The study is not powered to detect effects on major adverse cardiac events. As such, these analyses will only be exploratory.

Differences in outcomes between the control arm and intervention arm will be performed using an independent samples *t* test (continuous variables) and a chi-square test (categorical variables). If differences in baseline characteristics, number of follow-up visits, or telephone contacts are observed, linear and logistic regression analysis will be applied, adjusting for the observed differences between the two study arms.

A full study protocol and a Standard Protocol Items: Recommendations for Interventional Trials (SPIRIT) checklist (recommended items to address in a clinical trial protocol and related documents) are provided as additional files (Additional files 1 and 2).

## Discussion

Using the Internet and smartphones opens an array of new opportunities on how to provide care. It allows us to move away from a calendar-driven type of healthcare, where communication between patients and healthcare professionals is limited to booked appointments at a healthcare facility, to an individual-tailored type of care, where the patient can access information, receive advice independently of place and time, and take control over his or her treatment in a whole new way. Accordingly, the interest in eHealth solutions has grown exponentially during the last years, equally so from the industry, from patients, and from healthcare services. In the latest European Society of Cardiology guidelines on prevention in clinical practice, eHealth was advocated as a promising option for providing CR, while it was also pointed out that further studies in the field are needed [10]. We hope to contribute to this gap in evidence with the current study.

## Trial status

The inclusion of patients started in April 2016 and was finalized in April 2018, when all 150 patients had been included.

## Additional files

Additional file 1: Full study protocol with additional documents, including all questionnaires used in the study. (DOCX 587 kb)

Additional file 2: SPIRIT 2013 checklist: recommended items to address in a clinical trial protocol and related documents. (DOC 122 kb)

## Abbreviations

App: Web-based application
BMI: Body mass index
CCU: Coronary care unit
CHD: Coronary heart disease
CR: Cardiac rehabilitation
MI: Myocardial infarction
SEPHIA: Secondary Prevention after Heart Intensive Care Admission
SMS: Short text message
