# Supplementary material for: Effect of a lifestyle-focused electronic patient support application for improving risk factor management, self-rated health, and prognosis in post-myocardial infarction patients: study protocol for a multi-center randomized controlled trial
Source: Trials. 2019 Jan 24;20:76. doi: 10.1186/s13063-018-3118-1 (PMC6346565; doi:10.1186/s13063-018-3118-1)
Supplement: Supplementary file 1 — Full study protocol with additional documents, including all questionnaires used in the study. (DOCX 587 kb) [file 13063_2018_3118_MOESM1_ESM.docx]

# A multi-center, randomized, parallel-group, non-blinded study to compare the efficacy of a life-style focused electronic patient support application (app) and activity trackers for improving physical fitness, risk factor management, and prognosis in post-myocardial infarction patients

#

# Background

Cardiovascular disease (CVD) causes a large proportion of disability and death in Western countries. Only in Europe, it is responsible for close to 4.1 million deaths per year placing a substantial burden on the healthcare systems and economies (1). During the last decades, mortality rates from coronary heart disease (CHD) have decreased, with more than 50% of the mortality reduction attributable to better control of traditional cardiovascular risk factors (primary prevention) (2). When it comes to treatment of established disease, secondary prevention, administered through cardiac rehabilitation (CR), is the major contributor of the mortality reduction (3). CR includes specific core components comprising baseline patient assessment, nutritional counselling, risk factor management, psychosocial interventions, physical activity counselling and exercise (4). However, the current incomplete fulfilment of guideline recommended targets is a matter of concern. Sweden is not an exception. In the latest SWEDHEART report it was shown that one year after a myocardial infarction only 16% of patients reached the four main treatment objectives: abstinence from smoking, systolic blood pressure < 140 mmHg, low-density lipoprotein (LDL) < 1,8 mmol/l, and active participation in a structured exercise-based cardiac rehabilitation (CR) program (5).

It is well documented that participation in CR reduces recurrent events, improves risk factor control and therapy adherence and enhances quality of life (6). One main barrier to target achievement is limited accessibility to CP programs (7). Also, while international recommendations advocate program flexibility and individual tailoring, most of the current CR programs are rigid, time-limited and demand substantial healthcare resources (8). Therefore, all main international heart associations have claimed for the reengineering of CR to enhance access, adherence, and effectiveness. The general call is for the development of innovative and cost-effective rehabilitation programs oriented to modify lifestyle and behaviour with sustainable results and that may be easily integrated in the pre-existing healthcare structures (9).

eHealth

eHealth is a relatively recent term used to describe the combined use of electronic communication and health information technology in healthcare practice. eHealth solutions have been introduced in various fields of healthcare with promising results. Some examples include improving care for patients with long-term illnesses such as diabetes, remote monitoring of pacemakers and implantable cardioverter defibrillators and targeting negative health behaviours such as smoking. eHealth solutions have the advantage to overcome barriers of time and distance and potentially compensate for possible shortage of healthcare personnel in remote areas (10,11).

Using the internet and smartphones opens up an array of new opportunities on how to provide care. It allows us to move away from a calendar-driven type of healthcare, where communication between patients and healthcare personnel is limited to booked appointments at a healthcare facility, to an individual-tailored type of care, where the patient can access information, receive advice independent of place and time and take control over his or her treatment in a whole new way. Accordingly, the interest for eHealth solutions has grown exponentially during the last years, equally so from the industry, from patients as from healthcare services.

A few studies on using eHealth solutions, mostly using internet and smartphone-based interventions, for cardiac prevention and rehabilitation have been published (12-14). These have reported enhanced self-management of risk factors and that the interventions have the potential to improve well-being, decrease risk for recurrent events and increase adherence to medication, at a low cost (10-14). However, the majority of these studies have included only small numbers of patients with short follow-up (weeks or a few months) and have investigated a reduced number of surrogate end-points, limiting clinically useful conclusions. Furthermore only a small minority of available eHealth applications have been tested in a controlled manner with proper guidance from trained personnel. This is crucial as investment in new treatments and technologies in healthcare needs to be evidence-based (15,16).

Additionally, as the organization of healthcare systems for secondary prevention largely differs between countries, study design and conclusions are not directly applicable to the Swedish healthcare model.

Physical activity and accelerometers

Physical inactivity is a well-known risk factor for cardiovascular disease, based on general population studies, mostly using self-reports. Self-reports are often used in research due to their practicality, low cost and low participant burden. On the other hand, self-reports entail a risk of over- or underreporting, social desirability, as well as issues of recall and response bias (17,18). Presently, accelerometers are the method of choice for objective measurement of daily physical activity (18). Less is known about the importance of daily physical activity in secondary prevention, both as a prognostic marker and as a target for intervention. Studies are needed in order to determine the impact of different intensity levels of post-MI physical activity as well as sedentary time on cardiac recovery, risk factor control and prognosis.

# Aim & hypothesis

The general aim of the present study is improvement of post-MI secondary prevention, with focus on eHealth and exercise capacity/physical activity.

**Aim I. Use of eHealth solutions for improvement of adherence to secondary prevention**

The study will assess the efficacy of a web-based application as a complement to traditional CR for improvement of secondary prevention outcomes in post-MI patients, compared with usual care, using a multi-centre randomized controlled trial design. The hypothesis is that the intervention enhances patient adherence to lifestyle advice (exercise, daily physical activity, healthy diet and tobacco abstinence) and medication, resulting in better risk factor control, increased quality of life, improved cardiac recovery and prognosis.

**Aim II. Assessment and improvement of physical activity in MI patients by using activity trackers and accelerometers**

In half of patients in the intervention arm of the study described in Aim I physical activity will be assessed by using simple-to-use activity trackers, which feed directly to the web-based application. The activity tracking will be complemented by objective physical activity measurements with research-validated accelerometers at two time-points during follow-up. The hypothesis is that self-monitoring with the activity tracker will increase the interest for physical activity and adherence to guidelines. Additional hypothesis is that a moderate level of physical activity is related to improved metabolic control, cardiac recovery and prognosis.

# Methods

**Aim I. Use of eHealth solutions for improvement of adherence to secondary prevention**

The LifePod® platform

The LifePod® software is a web-based application designed to support persons in adhering to lifestyle advice and medication. The LifePod® system consists of two main parts, a patient interface (a smartphone or web-based application) and a medical interface managed by healthcare professionals. The medical interface allows for remote monitoring, facilitates patient prioritization and allocation of resources to where they are most needed.

**The patient interface**

The patient interface is a smartphone or web-based application (app), where the patient can log information about his/her lifestyle (i.e. diet, exercise, and smoking), measurements (i.e. weight, blood pressure, cholesterol, and blood glucose), symptoms, quality of life and medication. Screenshots of the patient interface can be seen in Figure 1. The patient can review his/her data in graphs displaying registered values in relation to guideline recommended targets. The patient unit gives the user immediate recommendations by flagging up poor food choices or insufficient physical activity, and to provide positive feedback on healthy choices. Additionally, the patient receives notices when the nurse at the clinic has reviewed his/her profile. The app generates reminders in the case of decreasing registrations or missed medication. Finally, short text messages (SMS) will be sent out 2-3 times a week with information and tips on healthy lifestyle. The interface can be customized, turning tiles and reminders on or off pending on needs and preferences of each patient.

**
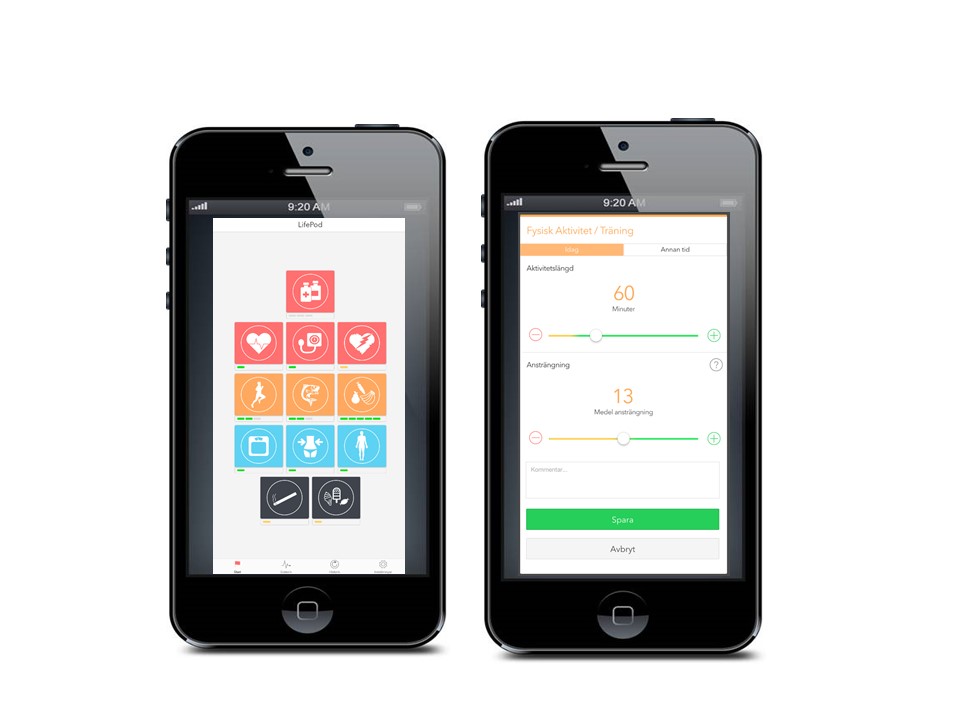
**

**Figure 1.** Screen shots of the LifePod 1.5 patient interface. The patient interface can be accessed through a smartphone, tablet or PC.

**The medical interface**The information that the patients log is transferred to a medical interface at the secondary prevention clinic. Via an algorithm the system ranks the patients according to need, giving high priority to patients for example reporting chest pains, are not taking their medications or lagged behind on their exercise regime, while patients that follow recommendations and are doing well receive lower ranking (figure 2). The information is reviewed by the CR nurse twice weekly. The algorithm in the LifePod® system thus gives priority support for the healthcare personnel based on patients’ reported outcomes. This allows for targeted interventions and better resource management. In addition, the system gives the caregiver the opportunity to manage and guide both individual patients (1:1 follow-up) and large patient populations (1:1000 or more).


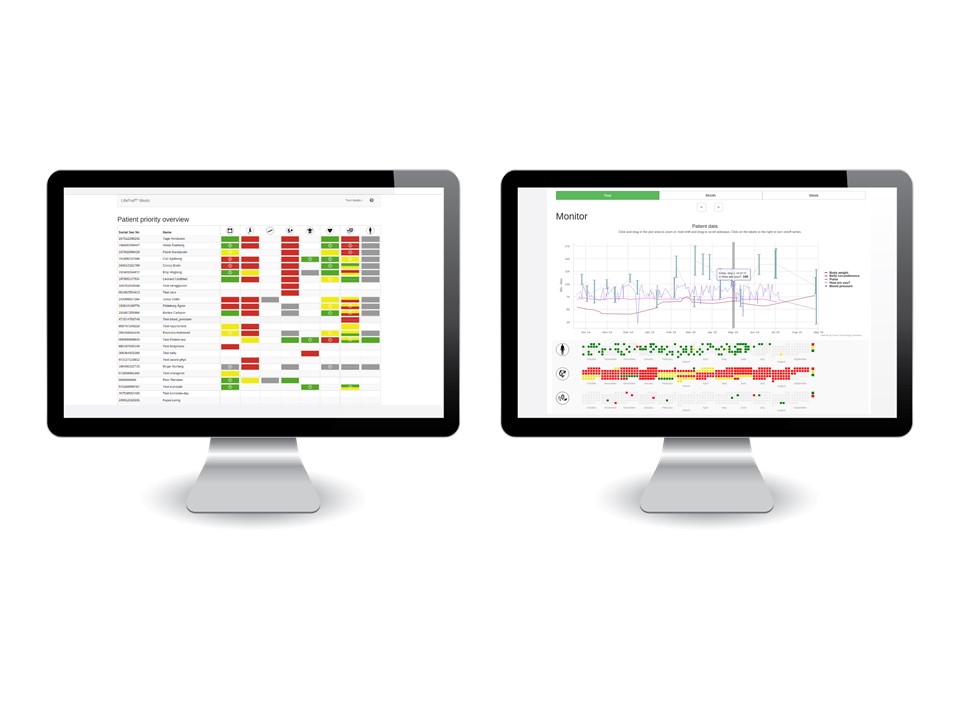


**Figure 2.** The LifePod 1.5 medical interface.

Study design, subjects and working plan

We will use an open-label, randomized, controlled, parallel group, superiority clinical trial design.

The aim is to recruit patients from at least three study centres:

1. Department of Coronary Care, Skåne University Hospital (SUS) in Malmö – primary study center
2. Department of Coronary Care, Skåne University Hospital (SUS) in Lund
3. Department of Cardiology, Norrland University Hospital (NUS) in Umeå

Approximately 1200 patients <75 years of age are treated for acute MI at the three study centres each year. These patients will form the basis for the project cohort. Patients will be screened for eligibility and offered participation in the study while admitted at the coronary care unit (CCU) for their index event (MI). Local study coordinators (physicians, nurses or physiotherapists, pending on convenience at each participating centre) will provide information about the study, offer participation and obtain a written informed consent. During admission, if the patients do not have their own smartphones, tablets (surf pads) will be provided at each recruiting site to enable patients to get acquainted with the software. Based on power calculations (see details below) the aim is to recruit 250 patients: 50 patients in the usual care arm, 100 patients in the LifePod arm and 100 patients in the LifePod+activity tracker arm (hereafter called LifePod+). The inclusion period is expected to last 2 years. Randomization will be done using sealed envelopes containing information on which group the patient is allocated to. The envelopes will be mixed by two persons after being sealed, after which they will be sequentially numbered.

**Inclusion criteria**

- Age < 75 years. This cut-off is set as only those < 75 years of age are followed in the national Secondary Prevention after Heart Intensive Care Admission (SEPHIA) registry
- Has suffered an MI within the last 2 weeks.
- Owns a smartphone and/or has access to Internet via a computer or surf pad and can handle the software

**Exclusion criteria**

- Expected survival < 1 year
- Dementia, severe psychiatric illness or drug abuse
- Severe physical handicap limiting the patient´s capacity to participate in a cardiac rehabilitation exercise program
- Planned coronary bypass surgery
- Not able to speak or understand the Swedish language

All patients will be encouraged to participate in CR according to the usual routine at each clinic, which can vary between sites. Patients randomized to the intervention arm will additionally receive the LifePod® support system and will be remotely monitored as described above. The patients in the intervention arm will have access to the LifePod® system for six months post MI. The study intervention will be discontinued if a participant withdraws consent. A study flow chart is shown in figure 3.

All patients will sign an informed consent. The study complies with the Declaration of Helsinki and the Swedish Research Council rules and guidelines for research.

Outcomes

All study centres routinely register data in the SWEDEHEART registry, both for the index event (RIKS-HIA) and for the cardiac rehabilitation phase (SEPHIA). Outcome measures will be extracted from the SEPHIA database, which is collected at several routine follow-up visits during the first year post-MI (Figure 3). Additionally, lipids, blood glucose, HbA1c, weight and blood pressure, self-reported dietary habits, physical activity and quality of life will be registered at approximately 6 months.


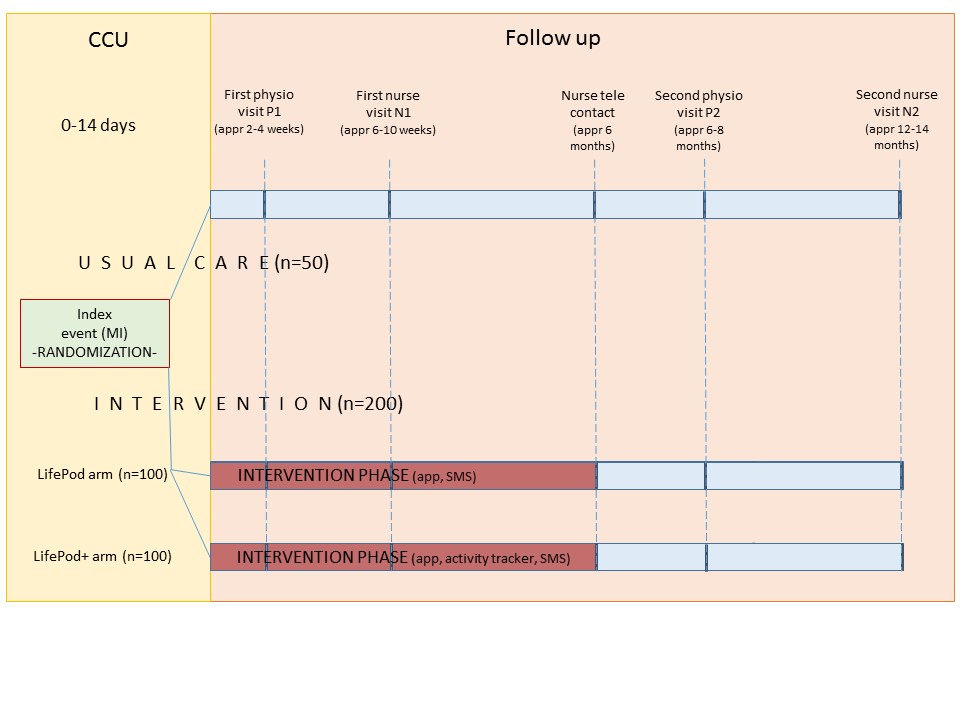


**Figure 3.** Flow chart for the study. N1: first nurse visit (conducted 6-8 weeks after the index event). N2: second nurse visit (conducted 12-14 months after the index event). P1: first physiotherapist visit (conducted before the patient starts exercise-based CR); P2: second physiotherapist visit (performed after the patient finishes the exercise-based CR). For variables registered at each visit see Appendix.

The efficiency of the eHealth intervention will be studied with regard to the following outcomes:

**Primary outcome**

The primary outcome, on which power calculations are based, is change in submaximal exercise capacity (W) between 2-4 weeks after discharge and when the patient has completed their exercise program at the CR center, usually around 3-6 months post-discharge. The submaximal exercise capacity reflects the patients´ level of fitness.

**Secondary outcomes**

1. Changes in the following variables measured from inclusion to first (6-8 weeks) and second (12-14 months) nurse visits, respectively:
   1. Self-rated health measured by Visual Analogue Scale (0-100)
   2. Healthy diet index (as measured within SEPHIA, see Appendix)
   3. Smoking habits
   4. Weight, BMI and waist circumference
   5. Systolic and diastolic blood pressure
   6. Cholesterol profile (total cholesterol, LDL, HDL, TG)
   7. Fasting glucose and HbA1c
2. Changes in self-reported physical activity (by Haskell, Frändin/Grimby and Visual Analogue Scale, see Appendix) measured from inclusion to first and second physiotherapist visits, respectively
3. Uptake (% of patients who log on to the patient interface at least once) and adherence (% of patients registering data at least twice a week) throughout the intervention period
4. Number of contacts with the CR staff
5. Incidence of cardiovascular events during the first year after the index event: hospitalization for a new MI, heart failure or stroke and cardiovascular death, measured at one and three years.

**Aim II. Assessment and improvement of physical activity in MI patients by using activity trackers and accelerometers**

Activity trackers

The activity trackers to be used in the LifePod+ study will be commercially available trackers (Figure 4). Basic activity trackers measure number of steps and distance covered using a built-in accelerometer. Retrospective analyses can be performed to analyse active vs. sedentary time during the day. Negotiations between Cross Solutions (the company behind LifePod) and several activity tracker producers are ongoing.


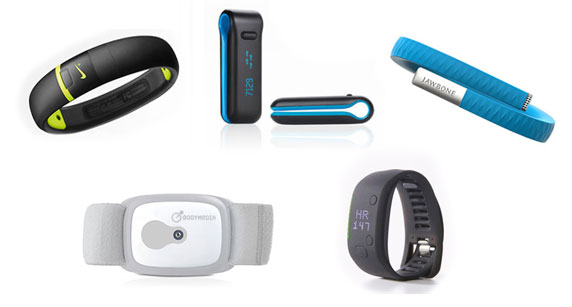


Figure 4. Different types of commercially available activity trackers.

Subjects and working plan

Patients in the intervention arm of the LifePod study in Malmö will be further randomized in two arms (100 patients in each), with patients in the LifePod+ arm being asked to wear the activity tracker for the duration of the intervention (6 months) as a complement to the LifePod app. The patients wearing the activity tracker will have the possibility to self-monitor their physical activity measured by the activity tracker through the LifePod app, which may lead to a higher awareness level and an improved training regime. In conjunction with the first and second physiotherapist visits (P1 and P2) 50 control patients, 50 LifePod patients and 50 LifePod+ patients, will undergo exact measurements of the duration and intensity of their physical activity, and of their sedentary time using ActiGraph accelerometers, for 7 consecutive days. ActiGraph accelerometers are validated instruments worn on the right hip that measure acceleration in the vertical plane (19). Correlations between self-reported activity levels, activity tracker-measured physical activity and ActiGraph data will be analysed, in order to assess whether the former two provide correct evaluations of physical activity compared to a validated instrument.

Comparisons will be made between patients using the app alone and a combination of app and activity tracker on changes in physical activity levels (by submaximal exercise capacity, self-reported levels of physical activity and ActiGraph measured activity). Also, both groups will be compared to usual-care controls on the same parameters.

Additionally, we will examine the effects of different levels of physical activity and sedentary time on levels of soluble biomarkers in plasma, cardiovascular risk factor control, improvement of systolic and diastolic heart function, as well as on the incidence of recurrent cardiovascular events during follow-up.

Statistical analysis/sample size calculations

Sample size calculations are based on results from a local pilot study (n=40) on differences in cardiac fitness measured by symptom-limited bicycle ergometer test before vs after particapting in a hospital-based exercise program as a part of cardiac rehabilitation. With a power of 90% and a two-sided significance level of 0.05 the estimated sample size is 250 patients: 50 patients in the usual care arm, 100 patients in the LifePod arm and 100 patients in the LifePod+ arm. This includes an intention-to-treat dilution effect based on a 40% loss of adherence in the intervention arms; estimation based on our feasibility study and other published studies measuring adherence to eHealth interventions in cardiac rehabilitation (12-14).

Baseline characteristics will be analysed using descriptive statistics (means, percentages). Differences between groups will be analysed using Student´s T-test (continuous variables) and Chi-square test (for categorial variables). All analyses will be done on an intention-to-treat basis. Only patients actively discontinuing the study will be excluded from the analyses.

# Preliminary results

At Skåne University Hospital (SUS) a non-randomized feasibility study to test the LifePod® application as a partial alternative to standard care recruited 50 patients between the fall 2013 and January 2015. Preliminary results show that 45% of the screened patients were included in the study. The main reasons for non-participation were lack of interest (43%), inability to manage the software (25%) and inadequate Swedish language skills (23%). Uptake, measured as the percentage of patients who logged on to the patient interface at least once was 40%. Adherence during the first 6 weeks, measured as the percentage of patients registering data at least twice a week on average during weeks 1-2, 3-4 and 5-6 was 82%, 63% and 56%, respectively. Main reasons for poor uptake and loss of adherence were finding the software too complicated, new illness, lack of time (especially after returning to work) and loss of motivation. Patients called for a more user-friendly interface, more positive feedback and implementation of sensors. Patients included in the feasibility study will be followed for one year.

The preliminary results from the feasibility study have been used to improve the software. The upgraded version is more simple and user-friendly, reminders have been implemented, patient feedback has been boosted and short messages with information on healthy lifestyle have been added. The medical interface has also been improved according to wishes from healthcare personnel involved in the feasibility study.

# Potential pitfalls

As the intervention can pose no harm to the participants there are no risks involved for the patients. Even though work is being done to improve the software based upon preliminary results from the feasibility study, there is of course a risk of poor adherence, as with all eHealth solutions, and thus a risk of negative results. In the case of poor adherence, complementary analysis will be made to analyse subgroups of adherent vs non-adherent subjects. Also, a retrospective qualitative post hoc analysis will be performed to further increase our understanding of non-adherence.

# Research environment

Patients will be recruited from the Coronary Care Units (CCU) at the participating hospitals. Study coordinators at each site will be responsible for the recruitment process. The patients will be followed at the CR units at the respective hospitals. The rehabilitation nurses and physiotherapists will be trained in supervising the medical interface. As such, no additional designated research facilities will be needed.

*Data collection and management*

Baseline data will be retrieved from RIKS-HIA (part of SWEDEHEART) and will include age, prior disease history and medication, type of MI, medical treatment, laboratory and physiological measures. In addition to variables included in RIKS-HIA, all patients will be asked to fill out questionnaires on diet, physical activity and self-rated health at baseline (see Appendix). Follow up/outcome data will be retrieved from the SEPHIA registry. Uppsala Centre of Research (UCR) is the registry administrating organizations for SWEDEHEART. Registry data quality is monitored centrally at UCR. All registry data as well as additional study data from questionnaires used in the study will be quality checked (i.e. duplicate measures, outliers, missing values) by the principal investigator (PI) and the researchers responsible for scientific analysis and writing. All of these have training in statistics and data management.

Primary responsibility for data monitoring will be assumed by the principal investigator (PI) and local study coordinators. Adherence to the intervention is monitored through the LifePod application, i.e. whether/ how often patients log on to the application and whether/how often the patients register data in the application. Adherence to usual care is monitored through the SWEDEHEART registry. Information about potential and enrolled participants will be collected by the local study coordinators. Outcome data will be collected by the PI. Files will be merged by the PI. Only the PI and members of her research team (PhD students and post-docs) will have access to the final trial dataset. The PI will oversee all data sharing, data integrity and data security. The PI takes primary responsibility for ensuring that the design of the study meets appropriate standards and that arrangements are in place to ensure appropriate conduct and reporting. The trial will be run in accordance with Good Clinical Practice (GCP) and all data will be handled according to Swedish Data Protection Regulations. As the majority of baseline and outcome data will be retrieved from a central registry we concluded that a data monitoring committee would not be necessary. No interim analysis will be done. As the intervention cannot cause harm to the participants adverse event monitoring will not be done. Audits will be done regularly by the local study coordinators.

*Ethical considerations*

The study protocol has been reviewed and approved Regional Ethical Review Board in Lund (approval number: 2016/5). The Regional Ethical Review Board will be informed about any study protocol amendments. Important protocol modifications will be communicated through clinicaltrials.gov and directly to the steering committee.

*Dissemination of results*

Results from the study will be presented at national and international scientific conferences and published in international peer-reviewed journals. Use of professional writers in not intended. The study protocol will be made public along with publication of a trial protocol manuscript.

The sponsor (Cross Solutions) will have full rights to use the published results to market their product. They will, however, not have access to study data to do own analyses.

Steering committee

Margret Leosdottir, PhD. Senior consulting Cardiologist at the Department of Coronary Care, Skåne University Hospital. Principal investigator. (*ML*)

Assoc Prof Alexandru Schiopu. Resident doctor in Cardiology at Skåne University Hospital. (*AS*)

Tina Tanha, PhD. Resident doctor in Cardiology at Skåne University Hospital. (*TT*)

Maria Bäck, PhD. Physiotherapist at Sahlgrenska University Hospital in Gothenburg and Senior University Lecturer at Linköping University. (*MB)*

Manuel Gonzalez, PhD. Consulting Cardiologist at the Department of Cardiology, Norrland University Hospital. (*MG*)

Ingela Sjölin. Physiotherapist and study coordinator at the Department of Coronary Care, Skåne University Hospital, Malmö and PhD student at Lund University. (*IS*)

Anneli Olsson, research nurse and study coordinator at the Department of Coronary Care, Skåne University Hospital, Lund. (*AO*)

Camilla Sandberg, PhD. Physiotherapist and study coordinator at the Department of Cardiology, Norrland University Hospital. (*CS*)

Funding

Under supervision of Dr. Leosdottir the rehabilitation team at Skåne University Hospital has worked closely with the company behind LifePod®, Cross Technology Solutions, to develop the software. The company has received financial support from Region Skåne to develop the software. The study is funded Lund University, the Department of Coronary Care at Skåne University Hospital and Cross Technology Solutions.

The funding sources had no role in the design of this study and will not have any role during its execution, analyses, interpretation of the data, or decision to submit results. The Steering committee delegates, including the PI and local study coordinators, have no conflicting interests to declare.

Sponsor contact information

The Department of Coronary Care at Skåne University Hospital:

Professor David Erlinge

The Department of Coronary Care at Skåne University Hospital

**Getingevägen 4, 222 41 Lund**

Email: [david.erlinge@med.lu.se](mailto:david.erlinge@med.lu.se); telephone: +46 46 17 10 00

Cross Technology Solutions:

Thomas Bergqvist (VD)

Åldermansgatan 10, 227 64 Lund

Email: [contact@cross-solutions.com](mailto:contact@cross-solutions.com); telephone: +46 700 40 04 02

# Protocol version

**Issue Date**: 18-10-17
**Protocol Amendment Number**: 02
**Author:** ML

**Revision Chronology:**

**2015-12-07: Original**

**2017-11-28: Amendment 01.** Primary reason for amendment: Adjusting protocol to SPIRIT and WHO standards

**2018-10-17: Amendment 02.** Primary reason for amendment: Revision av the study protocol for publication of protocol manuscript (addressing reveiwer´s comments).

# ****Authors’ contributions****

ML conceived of the study, coordinates the study and heads the steering committee. ML, AS, MB and BG initiated the study design. IS, AO and CS help with one-site implementation and are responsible for patient recruitment. *TT* is responsible for programming accelerometers and will supervise the LifePod+ study. MB provided statistical expertise in the clinical trial design. *ML,* IS, AO and CS are responsible for data quality monitoring. All authors contributed to refinement of the study protocol and approved the final protocol manuscript. Funding is administered by the Department of Coronary Care, Skåne University Hospital.

# World Health Organization Trial Registration Data Set

| **Data category** | **Information** |
| --- | --- |
| Primary registry and trial identifying number | ClinicalTrials.gov; NCT03260582 |
| Date of registration in primary registry | 24th of August 2017 |
| Source(s) of monetary or material support | Lund University |
| Primary sponsor | Lund University |
| Secondary sponsor(s) | Department of Coronary Care, Skåne University Hospital  Cross Technology Solutions |
| Contact for public queries | ML, IS. +4640331000; Margret.Leosdottir@med.lu.se |
| Contact for scientific queries | ML, IS. Department of Coronary Care, Skåne University Hospital |
| Public title | A life-style focused electronic patient support application (app) and activity trackers for improving physical fitness, risk factor management, and prognosis in post-myocardial infarction patients |
| Scientific title | A multi-center, randomized, parallel-group, non-blinded study to compare the efficacy of a life-style focused electronic patient support application (app) and activity trackers for improving physical fitness, risk factor management, and prognosis in post-myocardial infarction patients |
| Countries of recruitment | Sweden |
| Health condition(s) or problem(s) studied | Myocardial infarction |
| Intervention(s) | Access to a web-based application (app) designed to support persons in adhering to lifestyle advice and medication |
| Key inclusion and exclusion criteria | Ages eligible for study: 18-74 years Sexes eligible for study: both Accepts healthy volunteers: no Inclusion criteria:   - Age 18-74 years - Myocardial infarction within the last 2 weeks - Owns a smartphone and/or has access to Internet via a computer or surf pad and can handle the software   Exclusion criteria:   - Expected survival < 1 year - Dementia, severe psychiatric illness or drug abuse - Severe physical handicap limiting the patient´s capacity to participate in a cardiac rehabilitation exercise program - Planned coronary bypass surgery - Not able to speak or understand the Swedish language |
| Study type | Interventional Allocation: randomized Intervention model: parallel assignment Masking: none Primary purpose: secondary prevention Phase III |
| Date of first enrolment | April 2016 |
| Target sample size | 250 |
| Recruitment status | Recruiting |
| Primary outcome(s) | Change in submaximal exercise capacity (W) between 2-4 weeks and 3-6 months after discharge |
| Key secondary outcomes | Changes in self-reported physical activity, accelerometer-registered physical activity, self-rated health, dietary and smoking habits, body mass index, blood pressure, blood lipids, glucose/HbA1c levels between inclusion and follow-up visits during the first year post-MI, uptake and adherence to the application, the number of CR staff contacts and the incidence of cardiovascular events at one and three years. |

# References

1. Nichols M, Townsend N, Scarborough P and Rayner M. Cardiovascular disease in Europe: epidemiological update. Eur Heart J. 2013;34:3028-34.
2. Bjorck L, Rosengren A, Bennett K, Lappas G and Capewell S. Modelling the decreasing coronary heart disease mortality in Sweden between 1986 and 2002. Eur Heart J. 2009;30:1046-56.
3. Ford ES, Ajani UA, Croft JB*, et al.* Explaining the decrease in U.S. deaths from coronary disease, 1980-2000. N Engl J Med. 2007;356:2388-98.
4. Balady GJ, Williams MA, Ades PA, Bittner V, Comoss P, Foody JM, et al. Core components of cardiac rehabilitation/secondary prevention programs: 2007 update: a scientific statement from the American Heart Association Exercise, Cardiac Rehabilitation, and Prevention Committee, the Council on Clinical Cardiology; the Councils on Cardiovascular Nursing, Epidemiology and Prevention, and Nutrition, Physical Activity, and Metabolism; and the American Association of Cardiovascular and Pulmonary Rehabilitation. Circulation. 2007;115(20):2675-82.
5. Hambraeus K, Leosdottir M and Nilsson L. SEPHIA – Secondary prevention following coronary intensive care. SWEDEHEART Annual report 2014. Available on-line at: [www.swedeheart.se](http://www.swedeheart.se)
6. Wood DA, Kotseva K, Connolly S*, et al.* Nurse-coordinated multidisciplinary, family-based cardiovascular disease prevention programme (EUROACTION) for patients with coronary heart disease and asymptomatic individuals at high risk of cardiovascular disease: a paired, cluster-randomised controlled trial. Lancet. 2008;371:1999-2012.
7. Jackson L, Leclerc J, Erskine Y and Linden W. Getting the most out of cardiac rehabilitation: a review of referral and adherence predictors. Heart. 2005;91:10-4.
8. Perk J, De Backer G, Gohlke H*, et al.* European Guidelines on cardiovascular disease prevention in clinical practice (version 2012). The Fifth Joint Task Force of the European Society of Cardiology and Other Societies on Cardiovascular Disease Prevention in Clinical Practice (constituted by representatives of nine societies and by invited experts). Eur Heart J. 2012;33:1635-701.
9. Piepoli MF, Corra U, Adamopoulos S*, et al.* Secondary prevention in the clinical management of patients with cardiovascular diseases. Core components, standards and outcome measures for referral and delivery. Eur J Prev Cardiol. 2012.
10. Neubeck L, Redfern J, Fernandez R, Briffa T, Bauman A and Freedman SB. Telehealth interventions for the secondary prevention of coronary heart disease: a systematic review. Eur J Cardiovasc Prev Rehabil. 2009;16:281-9.
11. Pietrzak E, Cotea C, Pullman S. Primary and secondary prevention of cardiovascular disease: is there a place for internet-based interventions? J Cardiopulm Rehabil Prev. 2014;34(5):303-17
12. Varnfield M, Karunanithi M, Lee C-K *et al*. Smartphone-based home care model improved use of cardiac rehabilitation in postmyocardial infarction patients: results from a randomised controlled trial. Heart 2014;100:1770-1779.
13. Blasco A, Carmona M, Fernández-Lozano I et al. Evaluation of a telemedicine service for the secondary prevention of coronary artery disease. J Cardiopulm Rehabil Prev. 2012 Jan-Feb;32(1):25-31.
14. Piotrowicz E, Piotrowicz R. Cardiac telerehabilitation: current situation and future challenges. Eur J Prev Cardiol. 2013 Jun;20(2 Suppl):12-6.
15. Burke LE, Ma J, Azar KMJ, et al. Current science on consumer use of mobile health for cardiovascular disease: A scientific statement from the American Heart Association. Circulation 2015; DOI:10.1161/CIR.0000000000000232
16. Beatty AL, Fukuoka Y,. Whooley MA,. Mobile Technology for Cardiac Rehabilitation: A Review and Framework for Development and Evaluation. J Am Heart Assoc. 2013;2:e000568 doi: 10.1161/JAHA.113.000568.
17. Kohl HW 3rd. Physical activity and cardiovascular disease: evidence for a dose response. Med Sci Sports Exerc. 2001 Jun;33(6 Suppl):S472-83; discussion S493-4. Review. PubMed PMID: 11427773.
18. Sallis JF, Saelens BE. Assessment of physical activity by self-report: status, limitations, and future directions. Res Q Exerc Sport. 2000 Jun;71(2Suppl):S1-14.
19. Berntsen S, Hageberg R, Aandstad A, Mowinckel P, Anderssen SA, Carlsen KH, Andersen LB**: Validity of physical activity monitors in adults participating in free-living activities.** Br J Sports Med 2010, **44:**657-664.

# APPENDIX

# Variables to be registered at each time point

Index event

**From RIKS-HIA (national quality registry for acute myocardial infarction)**

Prior cardiovascular history (previous MI, stroke, PCI, CABG)

Diabetes (yes/no)

Smoking status

Medication at admission and discharge

Size (by TnT levels) and type of MI

Ejection fraction

Intervention performed in the acute phase

Height

Weight

Pulse, systolic and diastolic blood pressure

Lipid and glucose profile

**Additional study questionnaires**

Physical activity by Haskell (see below)

Healthy diet index (see below)

Quality of life by EQ-5D

First and second physiotherapist visits (P1 and P2)

Physical activity variables according to table below

Physical activity by Haskell

First and second nurse visits (N1 and N2)

Symptoms (angina and shortness of breath)

Diabetes (yes/no)

Smoking status

Medication

Acute re-admissions and planned interventions

Weight and waist circumference

Pulse, systolic and diastolic blood pressure

Lipid and glucose profile

Healthy diet index

Quality of life by EQ-5D

Nurse telephone contact (6 months)

Lipid and glucose profile

Systolic and diastolic blood pressure

Healthy diet index

Quality of life by EQ-5D

Physical activity by Haskell

Physical activity variables to be registered at first and second visits to physiotherapist

Resultat av cykeltest, muskelfunktion samt antal träningstillfällen vid start och stopp av fysisk träning inom hjärtrehabilitering

|  | **Test** | **Variabel** | **Enhet** | Min värde | maxvärde | Kommentar |  |
| --- | --- | --- | --- | --- | --- | --- | --- |
| Fysisk prestationsförmåga | Cykeltest ^RPE 15-17^ | Max Arbetskapacitet | Watt | 5 | 350 |  |  |
|  |  | Max watt | Minuter | 0,5 | 4,5 |  |  |
|  |  | Max Hjärtfrekvens | Slag per minut | 30 | 220 |  |  |
|  |  | Max Syst Blodtryck | mm Hg | 100 | 260 |  |  |
|  |  | Max Borg  Ansträngning | RPE |  |  | Grad på Borgs ansträngningskala  6-20 |  |
|  |  | Andfåddhet | CR10 | 0 | 12 | Grad på Borgs CR10 skala |  |
|  |  | Smärta | CR10 | 0 | 12 |  |  |
|  | Axelflexion | Max antal axelflexioner | Antal | 0 | 500 | Dominant arm  2 kg kvinnor  3 kg män |  |
|  | Tåhävning | Max antal hö | Antal |  |  |  |  |
|  |  | Max antal vä | Antal |  |  |  |  |

| Fysisk träning | Träningstillfällen på sjukhus/vårdcentral under rehabperioden | Sjukgymnastledd fysisk träning | Antal | 0 | 180 |  |  |
| --- | --- | --- | --- | --- | --- | --- | --- |
|  | Träningstillfällen på egen hand under rehabperioden | Sammanlagt minst 20 minuter med Borgskala 13-17 | Antal | 0 | 180 |  |  |
| Patientrapporterad  Fysisk prestationsförmåga | Begränsas du att utföra vad du önskar göra i vardagslivet av din nuvarande fysiska prestationsförmåga | Ja eller Nej | Ja = 1  Nej =2 | 1 | 2 |  |  |
| Patientrapporterad  Fysisk prestationsförmåga | Självupplevd fysisk prestationsförmåga | Visuell analog skala | mm | 0 | 100 |  |  |
| Patientrapporterad fysisk aktiviet Haskell sammanlagt 30 min/ dag | Fysisk aktivitet nivå | Likertskala  0-7 |  | 0 | 7 |  |  |
| Patientrapporterad  fysisk aktiviet Haskell sammanhängande minst 20 minuter per dag | Fysisk aktivitet nivå | Likertskala  0-7 |  | 0 | 7 |  |  |

Haskell´s questionnaire

Frändin/Grimby questionnaire

Healthy diet index

FRÅGEFORMULÄR OM MATVANOR

*Markera ett alternativ på varje fråga – det patienten brukar göra!*

**1. Hur ofta äter du grönsaker och/eller rotfrukter (färska, frysta eller tillagade)?**

Två gånger per dag eller oftare (3 p) ______

En gång per dag (2 p) ______

Några gånger i veckan (1 p) ______

En gång i veckan eller mer sällan (0 p) ______

**2. Hur ofta äter du frukt och/eller bär (färska, frysta, konserverade, juice etc.)?**

Två gånger per dag eller oftare (3 p) ______

En gång per dag (2 p) ______

Några gånger i veckan (1 p) ______

En gång i veckan eller mer sällan (0 p) ______

**3. Hur ofta äter du fisk eller skaldjur som huvudrätt, i sallad eller som pålägg?**

Tre gånger i veckan eller oftare (3 p) ______

Två gånger i veckan (2 p) ______

En gång i veckan (1 p) ______

Några gånger i månaden eller mer sällan (0 p) ______

**4. Hur ofta äter du kaffebröd, choklad/godis, chips eller läsk/saft**

Två gånger per dag eller oftare (0 p) ______

En gång per dag (1 p) ______

Några gånger i veckan (2 p) ______

En gång i veckan eller mer sällan (3 p) ______

**TOTALT: _____**

Ohälsosamma matvanor: 0-4 poäng Bra matvanor: 9-12 poäng

Self-rated health

**
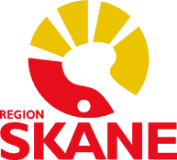
**

**Skriftlig patientinformation**

Implementering och evaluering av en web-baserad applikation (app)

och aktivitetsmätare för att förbättra levnadsvanor, riskfaktorer,

livskvalitet och prognos hos hjärtinfarktpatienter

Förkortat studienamn: LifePod studien

Forskningshuvudman: Region Skåne, Skånes Universitetssjukhus

Huvudprövarens namn: Margret Leosdottir

Kontaktnummer: 040 33 1000

Institution: Kranskärlskliniken

Skånes Universitetssjukhus

S-205 02 Malmö/S-221 85 Lund

Samarbetspartner:

Cross Technology Solutions

Åldermansgatan 10, SE-227 64 Lund, Sweden

Kontaktpersoner:

Thomas Bergquist, CEO

Maria Juul, CEO assistent

Kontaktnummer: 070 040 0402

**INLEDNING**

Du tillfrågas härmed om du vill delta i en klinisk forskningsstudie, då du har drabbats av en akut hjärtinfarkt inom de senaste 2 veckorna samt har tillgång till en s.k. smartphone eller dator/läsplatta. Studien heter LifePod studien. Studieläkaren har fastställt att du uppfyller de grundläggande kraven för att delta i studien. Innan du samtycker till att delta i studien är det viktigt att du läser nedanstående beskrivning. Ta dig tid att ställa så många frågor som du vill. Studiepersonalen kommer att förklara ord eller information som du inte förstår. I dokumentet beskrivs studiens syfte, hur studien går till, möjliga fördelar samt risker. Här beskrivs också din rättighet att när som helst välja att inte delta i studien. Du behöver inte förklara varför om du beslutar dig för att inte delta eller om du avbryter ditt deltagande.

Studien utförs av Kranskärlskliniken, Skånes Universitetssjukhus, i samarbete med Cross Technology Solutions. Studien har granskats och godkänts av Läkemedelsverket och den Regionala Etikprövningsnämnden.

**SYFTET MED STUDIEN**

Syftet med LifePod studien är att undersöka om en interaktiv web-baserad applikation (app, se Bild 1) kan påverka följsamhet till livsstilsråd och medicinering och i förlängningen måluppfyllelse avseende levnadsvanor, riskfaktorer och livskvalitet hos hjärtinfarktpatienter, jämfört med sedvanlig vård. Appen kan nås via en internet-uppkopplad smart telefon, en dator eller surfplatta. En subgrupp av deltagare kommer utöver appen få en aktivitetsmätare (kallas också smart armband) som komplement till appen. Aktivitetsmätaren ger återkoppling till patienten om hans/hennes dagliga fysiska aktivitetsnivå.

**
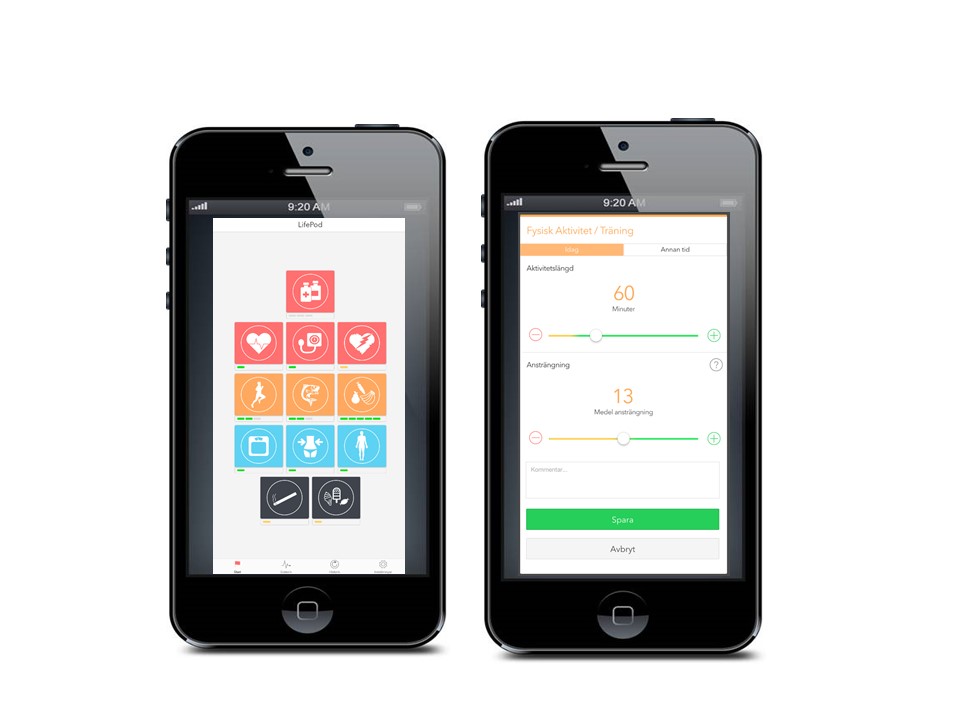
**

Bild 1. Skärmbilder från LifePod appen.

**HUR GÅR STUDIEN TILL?**

Studien utförs i Sverige och ca 250 patienter på olika sjukhus i landet kommer att delta.

Om du samtycker kommer du att lottas till en av tre studiegrupper:

1. Jämförelsegrupp. Denna grupp får sedvanlig uppföljning på Kranskärlsmottagningen enligt klinikens rutiner. I flesta fall följs man på mottagningen i ett år, med slutbesök till sköterska när det har gått 12-14 månader efter hjärtinfarkten. Du kommer att får mer noga information om uppföljningen vid hemskrivning från sjukhuset.
2. App-grupp. Denna grupp får sedvanlig uppföljning såsom ovan, men får även tillgång till LifePod appen i sex månader efter hjärtinfarkten.
3. App + ativitetsmätargrupp. Denna grupp får sedvanlig uppföljning såsom ovan, tillgång till LifePod appen samt en aktivitetsmätare i sex månader efter hjärtinfarkten.

Om du lottas till jämförelsegruppen ombeds du svara på ett par korta frågeformulär när du inkluderas i studien, samt samma formulär under uppföljningsbesöken på Kranskärlsmottagningen. Annars kommer uppföljningen att löpa på som vanligt och inget ytterligare krävs av dig.

Om du lottas till app-gruppen hjälper forskningspersonalen dig att ladda ner LifePod appen i din smartphone. Web-adress till appen kommer du också att få så att du kan logga in på appen på din dator eller läsplatta hemma. Du kommer att ha tillgång till appen under de första 6 månaderna efter hjärtinfarkten. Via appen kan du registrera dina levnadsvanor (i.e. kost och motion), värden (i.e. vikt, midjemått och blodtryck) och medicinering. Du får också stöd och råd i form av återkoppling på inmatade värden, informationstexter i appen samt regelbundna SMS. Du kan via appen se trender och se hur du ligger till i förhållande till de hälsoråd som följs på kliniken. Appen rapporterar informationen som du matar in till din sköterska på mottagningen som via ett medicinskt gränssnitt har chans att reagera på larmvärden men framförallt får en bild av hur du ligger i dina värden och vanor inför återbesök på mottagningen. Fördelen med detta system antas vara att en mer kontinuerlig egen monitorering och återkoppling avseende dina levnadsvanor leder till en gynnsammare hälsoprofil och minskad hjärtkärlsjuklighet.

Om du lottas till app+aktivitetsmätare får du utöver appen en aktivitetsmätare i form av ett armband, som registrerar din aktivitet, såsom antal steg per dag, aktivitetsgrad vid träning etc. Såsom för appen kommer du att ha tillgång till aktivitetsmätaren i 6 månader och uppmuntras ha den på dig varje dag.

Ett mindre antal patienter i varje grupp kommer att ombes bära på ytterligare en validerad aktivitetsmätare som man har i ett bälte runt midjan i några dagar, två gånger under uppföljningen. Syftet med denna mätning är få en mer detaljerad mätning av patienternas aktivitetsnivå samt för att se om det smarta armbandet ger en rättvis bild av uppmätt aktivitet.

För samtliga patienter kommer vi att hämta hem data från patientjournaler och centrala register (Sveriges kvalitetsregister för hjärtsjukvård, SWEDEHEART, samt diagnosregister). Data som samlas från dessa källor inkluderar information om tidigare hälsohistoria, nuvarande hjärtinfarkt, uppmätta parametrar under uppföljning (inklusive blodtryck, kolesterol, vikt, kostvanor, fysisk prestationsförmåga, medicinering och livskvalitet) samt återinsjuknande. Dessa data kommer att användas för att utvärdera effekten av appen och aktivitetsmätaren.

Det är viktigt att du förstår att ditt deltagande i studien kräver ett 6-12 månader långt åtagande från dig genom att komma på planerade återbesök samt om du lottas till app-gruppen (+/- aktivitetsmätare) att du använder appen och aktivitetsmätaren enligt anvisningar.

**MÖJLIGA FÖRDELAR**

De patienter som lottas till app +/-aktivitetsmätare kommer att få utökat stöd för att hantera sina levnadsvanor och riskfaktorer, jämfört med sedvanlig vård. Man kommer dessutom att distansövervakas av kranskärlssköterska via det medicinska gränssnittet och således ha en ytterligare kommunikationskanal med mottagningen utöver det sedvanliga. Ytterligare så är förhoppningen att appen leder till ett lärande om vikten av följsamhet till hälsosam livsstil och medicinering framöver, som gagnas dig framöver.

**FINNS DET NÅGRA RISKER ELLER OBEHAG?**

Några uppenbara risker med studien har inte kunnat identifieras.

**BIOBANKSPROVER**

Inga prover förutom de prover som tas som enligt rutin i den vanliga vården kommer att tas.

**HANTERING AV DATA OCH SEKRETESS**

Data som du matar in i appen samt data från aktivitetsmätarna samlas in till en server som sköts av Cross Solutions. Samtlig kommunikation är krypterad till och från appen/aktivitetsmätaren och det medicinska gränssnittet som mottagningssköterskorna har tillgång till. Samtlig access till systemet är via individuella licenser med autentisering (lösenord och användarnamn). På företagets sida är data krypterad och accessen reglerad på liknande sätt. Företagets servrar står i skyddat före detta militärt bergrum i Sverige. Företagets personal kommer inte att ha tillgång till register- eller journaldata. Data som hämtas från journal och centrala register är skyddat av sekretess och endast din mottagningssköterska och studieansvariga har tillgång till dessa. All involverad personal har tystnadsplikt. Enligt gällande sekretessbestämmelser kommer svar och resultat att behandlas så att obehöriga inte får tillgång till dem (beskrivs på www.skane.se/informationssakerhet).

**INFORMATION OM STUDIENS RESULTAT**

Studiens resultat kommer att meddelas alla deltagare efter studiens slut. Resultaten kommer att publiceras i vetenskapliga tidskrifter och på möten, men information om enskilda patienter går inte att identifiera.

**FÖRSÄKRING OCH ERSÄTTNING**

Deltagare i studien omfattas av den generella patientskadeförsäkringen. Någon ersättning för deltagande i studien ingår inte.

**FRIVILLIGHET**

Deltagandet i studien är helt frivilligt och du kan, även efter att du gett ditt godkännande, när som helst ta tillbaka detta om du så önskar utan att det påverkar vården.

**RÄTTIGHETER ENLIGT PERSONUPPGIFTSLAGEN**

Du har rätt enligt Personuppgiftslagen (Pul) att ansöka om information från personuppgiftsombudet, Region Skåne 291 89 Kristianstad. En sådan ansökan måste vara undertecknad av dig som patient. Man kan i en sådan ansökan begära att få uppgifter rättade som är felaktiga.

**FRÅGOR KRING STUDIEN**

Du kan ställa frågor kring studien till studieansvarig läkare:

Margret Leosdottir

Kranskärlskliniken, Skånes Universitetssjukhus.

Telefon 040 33 1000

Margret.Leosdottir@med.lu.se

Du kan också ställa frågor kring mjukvaran (appen och aktivitetsmätaren) som används i studien direkt till företaget, antingen telefonledes eller via konkaktformulär på hemsidan [www.lifepod.se](http://www.lifepod.se).

Cross Technology Solutions

Åldermansgatan 10

SE-227 64 Lund

Kontaktperson:

Maria Juul, CEO assistent

Telefon 070 040 0402

*Patientens exemplar*

INFORMERAT SAMTYCKE

Om du har frågor om studien eller om detta dokument bör du ställa dem innan du undertecknar formuläret. Din studieläkare/sköterska kommer att försöka svara på alla frågor du har så fullständigt som möjligt, före, under och efter studien.

Undertecknad har läst (eller har lästs upp för mig) informationen i detta dokument. Jag har haft tillräckligt med tid för att sätta mig in i syftet, procedurerna och möjliga risker och fördelar med studien.

Jag har rätt att dra mig ur studien när som helst, av vilket skäl som helst, och beslutet att inte delta kommer inte att påverka min framtida medicinska vård.

*Underteckna endast detta samtyckesformulär om alla följande påståenden är sanna:*

- Informationen i detta dokument har förklarats för mig och jag har fått tillfälle att ställa frågor.
- Jag samtycker till att min medicinska information samlas in och används såsom beskrivs ovan.
- Jag som lottas till intervention (app +/- aktivitetsmätare) samtycker till att mina data som jag matar in i appen och mäts i aktivitetsmätaren förvaras och bearbetas av Cross Technology Solutions eller deras representanter.
- Jag har beslutat att delta i denna forskningsstudie och jag kommer att få en undertecknad och daterad kopia av detta dokument.

_____________________________________

Patientens namn (texta)

_____________________________________ _______________________

Patientens underskrift Datum för underskrift

Undertecknad prövare/underprövare, bekräftar att jag muntligen har informerat om studien, att jag besvarat alla ytterligare frågor, och att jag inte utövade något tryck på patienten att delta i studien.

Jag intygar att jag handlade helt i enlighet med de etiska principer som beskrivs i GCP-riktlinjerna och andra gällande nationella och internationella lagar.

Patienten kommer att få en kopia av denna skriftliga patientinformation och samtyckesformulär, som undertecknats av båda parter.

_____________________________________

Prövarens namn (texta)

_____________________________________ _______________________

Prövarens underskrift Datum för underskrift

*Studiepersonalens exemplar* ID.nr: _____________

INFORMERAT SAMTYCKE

Om du har frågor om studien eller om detta dokument bör du ställa dem innan du undertecknar formuläret. Din studieläkare/sköterska kommer att försöka svara på alla frågor du har så fullständigt som möjligt, före, under och efter studien.

Undertecknad har läst (eller har lästs upp för mig) informationen i detta dokument. Jag har haft tillräckligt med tid för att sätta mig in i syftet, procedurerna och möjliga risker och fördelar med studien.

Jag har rätt att dra mig ur studien när som helst, av vilket skäl som helst, och beslutet att inte delta kommer inte att påverka min framtida medicinska vård.

*Underteckna endast detta samtyckesformulär om alla följande påståenden är sanna:*

- Informationen i detta dokument har förklarats för mig och jag har fått tillfälle att ställa frågor.
- Jag samtycker till att min medicinska information samlas in och används såsom beskrivs ovan.
- Jag som lottas till intervention (app +/- aktivitetsmätare) samtycker till att mina data som jag matar in i appen och mäts i aktivitetsmätaren förvaras och bearbetas av Cross Technology Solutions eller deras representanter.
- Jag har beslutat att delta i denna forskningsstudie och jag kommer att få en undertecknad och daterad kopia av detta dokument.

_____________________________________

Patientens namn (texta)

_____________________________________ _______________________

Patientens underskrift Datum för underskrift

Undertecknad prövare/underprövare, bekräftar att jag muntligen har informerat om studien, att jag besvarat alla ytterligare frågor, och att jag inte utövade något tryck på patienten att delta i studien.

Jag intygar att jag handlade helt i enlighet med de etiska principer som beskrivs i GCP-riktlinjerna och andra gällande nationella och internationella lagar.

Patienten kommer att få en kopia av denna skriftliga patientinformation och samtyckesformulär, som undertecknats av båda parter.

_____________________________________

Prövarens namn (texta)

_____________________________________ _______________________

Prövarens underskrift Datum för underskrift
